# Fluorescent Cell Barcoding as a Tool to Assess the Age-Related Development of Intracellular Cytokine Production in Small Amounts of Blood from Infants

**DOI:** 10.1371/journal.pone.0025690

**Published:** 2011-10-17

**Authors:** Jose Stam, Wayel Abdulahad, Minke G. Huitema, Caroline Roozendaal, Pieter C. Limburg, Margriet van Stuijvenberg, Elisabeth H. Schölvinck

**Affiliations:** 1 Beatrix Children's Hospital, University Medical Centre Groningen, Groningen, the Netherlands; 2 Department of Rheumatology and Clinical Immunology, University Medical Centre Groningen, Groningen, the Netherlands; 3 Department of Laboratory Medicine, University Medical Centre Groningen, Groningen, the Netherlands; Agency for Science, Technology and Research - Singapore Immunology Network, Singapore

## Abstract

Fluorescent Cell Barcoding (FCB) is a flow cytometric technique which has been used for assessing signaling proteins. This FCB technique has the potential to be applied in other multiparameter analyses. Since data on antigen (Ag)-specific T-cell immune responses, like intracellular cytokine production, are still lacking in infants because limited blood volumes can be obtained for analysis, the FCB technique could be very useful for this purpose. The objectives of this study were to modify the FCB method to be able to measure multiple Ag-specific cytokine reponses in T-cells upon simultaneous stimulation by various antigens and mitogens in small amounts of blood and to investigate the cytokine pattern of T-cell subsets in healthy infants aged six and twelve months. Blood samples, collected from 20 healthy infants aged six and twelve months, were stimulated *in vitro* with the antigens: phorbol-myristate-acetate (PMA), purified-protein-derivative (PPD), Tetanus-toxoid (TT), Staphylococcal-enterotoxin-B (SEB), and phytohemagglutinin (PHA). Each stimulus was barcoded by labelling with different intensities of fluorescent cell barcoding (FCB) markers. Intracellular production of interleukin-2, interferon-gamma, and tumor necrosis factor-alpha was measured simultaneously in just one blood sample of 600 µl whole blood. Significant age-related differences in cytokine production were shown for PMA, PHA, and TT in CD4^+^ T-cells, and for PMA, PHA, SEB, and TT in CD8^+^ T-cells. The intracellular cytokine production by CD4^+^ and CD8^+^ T-cells was higher at twelve months compared to six months of age for all antigens, except for PMA, which was lower at the age of twelve months. Based on the consistency in both T-cell subsets, we conclude that the new FCB method is a promising tool to investigate the age-related development of intracellular cytokine production in infants.

## Introduction

T-cells are the cornerstone of the adaptive immune system and play an essential role in the host defense against microbial pathogens [1]. Their responses are commonly recognized by the secretion of various cytokines, such as interleukin-2 (IL-2), interferon-gamma (IFN-γ) and tumor necrosis factor-alpha (TNF-α) [2]–[4]. By analyzing cytokine patterns of T-cells, the distinction between normal and abnormal immune responses can be made under various conditions. Previous reports have described methods, such as ELISA and ELISPOT, to identify *ex-vivo* cytokine expression by antigen-stimulated peripheral blood mononuclear cells (PBMCs). However, these methods cannot provide information on the frequency and the phenotype of antigen (Ag) -responsive cells. Flow cytometry has been developed to assess Ag-specific T-cell responses by analyzing intracellular cytokine expression, and has proven to be more sensitive and informative than traditional methods [5]–[7].

Quantifying Ag-induced T-cell responses by flow cytometry in previous studies was restricted to samples from adults, whereas little is known on reference values and age-related differences of T–cell immune responses in infants. One of the restraints in gaining knowledge on this issue has been the need for substantial amounts of blood in traditional investigatory methods. Recently, a new flow cytometric technique, called fluorescent cell barcoding (FCB), was developed to measure the phosphorylation status of proteins critical to intracellular cascades at the single-cell level in small samples of blood [8]. Modifying the FCB technique for analyzing intracellular cytokine expression of T-cells may be useful for young children where limited blood volumes can be obtained for analysis.

To this end, the first objective of our study was to modify the previously described FCB method to be able to measure multicytokine responses in T-cells, upon simultaneous stimulation by various antigens and mitogens, in limited amounts of blood. Since few studies have looked systematically at age-related differences in healthy children [9]–[12], our second objective was to compare the intracellular cytokine pattern of three pivotal Th1-type cytokines in healthy infants aged six and twelve months using the modified FCB method.

## Materials and Methods

### Subjects and sample handling

This study was imbedded in a larger prospective multicenter nutritional intervention study. The details of the full study setup have been described elsewhere [13]. In summary, healthy term infants were included. Infants were excluded if they had a family history of atopic or other immune-mediated diseases, congenital diseases, or neonatal complications. All infants were immunized according to Dutch the national immunization program.

In our study, lymphocyte subpopulations (total lymphocyte number, CD3^+^, CD4^+^, CD8^+^ T-cells, and CD19^+^ B-cells, and CD16^+^56^+^ NK-cells) as well as the intracellular production of three pivotal Th1-type cytokines (IL-2, IFN-γ, and TNF-α) in CD4^+^ and CD8^+^ T-cell subsets were assessed in 20 healthy infants born in the Netherlands of Dutch parents.

Heparinized venous blood was collected under sterile conditions, stimulated and incubated within three hours after collection. Blood from each individual infant was drawn twice, at six and twelve months of age. The study was approved by the Medical Ethics Review Board of the University Medical Centre Groningen. Written informed consent was obtained from all caregivers (Trial registration: German Clinical Trials Register DRKS00000201).

### Lymphocyte subset enumeration

Lymphocyte subsets (total lymphocyte number, CD3^+^, CD4^+^, CD8^+^ T cells, CD19^+^ B-cells, and CD16^+^56^+^ NK-cells) were measured as absolute numbers using the Becton Dickinson MultiTest TruCount method with four-color MultiTest reagents CD3/8/45/4 and CD3/16+56/45/19 (Becton Dickinson). The lyse-no-wash preparation method was performed as described by the manufacturer. Flow cytometry was performed on FACSCalibur (Becton Dickinson) and analysis was performed using Attractors software (Becton Dickinson).

### Sample preparation and in vitro stimulation

Heparinized venous blood was obtained from 20 infants. Within three hours after sampling, 600 µl blood was mixed with 600 µl RPMI1640 (Cambrex Bio Science, Verviers, Belgium), supplemented with 50 µg/ml gentamycin (Gibco, Scotland, UK), and aliquoted into 6 polypropylene tubes (Becton Dickinson; 200 µl per tube). In 4 of the 6 tubes, diluted blood samples were stimulated with either 0.5 TE/ml purified protein derivative (PPD; Netherlands Vaccine Institute (NVI), Bilthoven, The Netherlands), 15 Limit of flocculation units (Lf)/ml Tetanus toxoid (TT; NVI), 5 µg/ml Staphylococcal enterotoxin B (SEB; Sigma, Deisenhofen, Germany), or 5 µg/ml phytohemagglutinin (PHA; Remel, Lenexa, KS, USA). One control sample remained without stimulation. To provide optimal co-stimulation, co-stimulatory reagent (anti-CD28/anti-CD49d; Becton Dickinson) was added to each tube at 1 µg/ml. Samples were incubated at 37°C and 5% CO_2_. After 2 hours of stimulation, 10 µg/ml brefeldin A (Sigma-Aldrich, Zwijndrecht, The Netherlands) was added to each tube in order to disrupt protein transport to allow for intracellular accumulation of cytokines. Next, culture tubes were incubated for a total of 24 hours. To determine the total level of cytokine secretion, one of the diluted blood samples was stimulated with 40 nM phorbol myristate acetate (PMA; Sigma-Aldrich) and 2 nM calcium ionophore A23187 for four hours (Sigma-Aldrich) in the presence of 10 µg/ml brefeldin A.

### Fluorescent cell barcoding (FCB) and immunofluorescent staining of cell subpopulations

**Figure 1:**
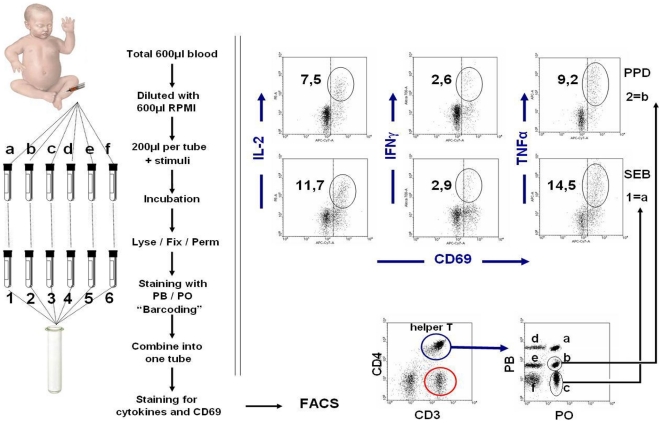
Schematic representation of the quantification of cytokine secreting T-cells in whole blood from an infant via fluorescent cell barcoding (FCB) technique. Blood sample was diluted with RPMI, and divided into 6 tubes and stimulated as described in materials and methods. After incubation, samples were lysed/fixed/permeabilized and stained with different concentration of Pacific Blue (PB) and Pacific Orange (PO) dyes, yielding a unique fluorescence signature for each sample. Next, samples were washed, combined into one tube, and simultaneously stained with fluorochrome conjugated antibodies as given in Materials & Methods. As indicated, samples were measured on FACS and CD4^+^ or CD8^+^ T-cells were gated and analyzed for their FCB fluorescence intensity to identify the sample origin of cells. Cells within each gate were analysed for the expression of the activation marker CD69 versus intracellular cytokine production of IL-2, IFN-γ, and TNF-α.

The procedure of the modified FCB method is displayed in Figure 1. Following incubation, samples were pre-treated with 10 µl of 40 mM EDTA in PBS for 10 minutes to prevent adhesion of activated cells. Next, red blood cells were lysed and white blood cells fixed by adding 2 ml of FACS lysing solution (Becton Dickinson) for 10 minutes at room temperature, and cells were then washed in PBS containing 1% bovine serum albumin (BSA). Subsequently, each sample was permeabilized in 500 µl of Perm II (Becton Dickinson) containing a different concentration and/or combination of Pacific Blue (PB) and/or Pacific Orange (PO) dyes (Invitrogen, Carlsbad, CA, USA) to enable fluorescent cell barcoding (FCB) of each original tube as described in Figure 1. Unstimulated samples were stained with 5 µg PB+10 µg PO, whereas samples stimulated with PPD, SEB, TT, PHA, and PMA were stained with 1,25 µg PB+10 µg PO, 0 µg PB+10 µg PO, 10 µg PB+0 µg PO, 1,25 µg 0 µg PO, and 0 µg PB+0 µg PO, respectively. After incubation for 10 minutes at room temperature in the dark, samples were washed and resuspended in PBS containing 20% fetal calf serum (FCS).

Next, the different FCB-samples were combined into one FACS-tube, then washed, resuspended in 100 µL of wash buffer, and stained with the following antibodies: 7,5 µL of FITC-anti-CD3 (clone SK7), 7,5 µL of PE-Cy7-anti-CD4 (clone SK3), 7,5 µL of PerCP-anti-CD8 (clone SK1), 7,5 µL of APC-Cy7-anti-CD69 (clone FN50), 4 µL of APC-anti-TNF-α (clone Mab11), 7,5 µL of PE-anti-IL-2 (clone MQ1-17H12), and 7,5 µL of Alexa700-anti-IFN-γ (clone B27). All antibodies were purchased from Becton Dickinson (Breda, The Netherlands). We titrated all conjugates for optimal solution and saturating concentration. Labelled samples were incubated for 30 minutes at room temperature in the dark. After staining, the cells were washed twice, resuspended in 750 µl PBS/1%BSA, and immediately analyzed on FACS-LSRII flow cytometer (Becton Dickinson). Most of the dead cells were washed out during the several wash steps in the procedure.

### Flow cytometric analysis

Nine-colour flow cytometric acquisition was performed on LSR-II using FACS-Diva software (Becton Dickinson). For all flow cytometry analyses, data were collected for at least 1×10^6^ cells, and plotted using the Win-List software package (Verity Software House Inc, ME, USA). First, lymphocytes were gated by CD3^+^ expression and sideward scatter patterns, and divided into CD4^+^ and CD8^+^ T-cell populations. Next, cells from different stimulation tubes were identified based on their FCB signature (as shown in Figure 1), gated separately, and analysed as individual samples for the expression of the activation marker CD69 versus intracellular cytokine production of TNF-α, IL-2, and IFN-γ. Unstimulated samples were used for setting the linear gates to delineate positive and negative populations. Results were expressed as the percentage of cytokine-producing CD69^+^ cells within the total CD4^+^ or CD8^+^ T-cell population. Values were corrected for unstimulated cultures. Dead cells not washed out by the several wash steps of the FCB procedure were further excluded from the lived gate according to their forward- and side-scatter pattern.

### Statistical analysis

Statistical analysis was carried out using SPSS software Version 16.0. The non-parametric Wilcoxon signed ranks test for paired values was used to compare median values of intracellular cytokine production of the same infants at the age of six and twelve months. A p-value<0.05 was considered significant.

## Results

### Subjects

Twenty healthy, term infants born in the Netherlands were recruited for this study. Their characteristics are shown in Table 1. All infants were immunized at the age of two, three, and four months, as well as at eleven months according to the Dutch national immunization program before the blood sample was drawn at the age of six and twelve months, respectively.

**Table 1:** Subject characteristics of 20 healthy infants born in the Netherlands.

Category	Subcategory	Outcome
Sex	Male	6 (30)
	Female	14 (70)
Education mother	Primary and secondary school	8 (40)
	Some university or postsecondary education	4 (20)
	Technical or trade qualification	8 (40)
Education father	Primary and secondary school	6 (30)
	Some university or postsecondary education	5 (25)
	Technical or trade qualification	9 (45)
Delivery	Vaginal	15 (75)
	Caesarean	5 (25)
Feeding regimen	Formula feeding	18 (90)
	Breastfeeding	2 (10)
Twins		4 (40)
Birth weight, gram		3135 (2560–5150)
Gestational age, weeks		39 (37–42)
Age of the mother, years		29.5 (19–37)

- Categorical variables are presented as number (percentages).

- Numerical variables are presented as median (range).

### T-cell subpopulations

The absolute numbers of total lymphocytes and CD3^+^, CD4^+^, CD8^+^ T-cells, CD19^+^ B- cells, and CD16/56^+^ NK-cell subpopulations measured at the age of six and twelve months are given in Table 2. A significant lower number of total lymphocytes, CD3^+^, CD4^+^, and CD19^+^ cells was observed at 12 months compared to six months of age. No significant differences were found for the number of CD8^+^ and CD16/56^+^ NK-cells.

**Table 2:** Values of lymphocyte subpopulations in peripheral blood (numbers ×10^9^/l).

Subpopulations	Subcategory	Age 6 months	Age 12 months	p-value^b^
		(n = 20)^a^	(n = 20)^a^	
Total lymphocytes	Median	9.4	6.1	0.001
	Range	(8.5–11.1)	(5.0–7.2)	
CD3^+^	Median	5.0	4.5	0.007
	Range	(4.5–6.9)	(2.9–5.0)	
CD4^+^	Median	3.7	3.2	0.003
	Range	(3.4–4.4)	(1.8–3.4)	
CD8^+^	Median	1.2	1.0	n.s.
	Range	(0.9–2.2)	(0.8–1.5)	
CD19^+^	Median	1.7	1.4	0.029
	Range	(1.4–2.1)	(0.9–1.8)	
CD16/56^+^	Median	0.3	0.3	n.s.
	Range	(0.3–0.4)	(0.2–0.6)	

a Median (25–75^th^ percentiles).

b Significance level 0.05.

### The frequency of CD69^+^-expression in CD4^+^ and CD8^+^ T-cells upon exposure to stimuli

**Figure 2:**
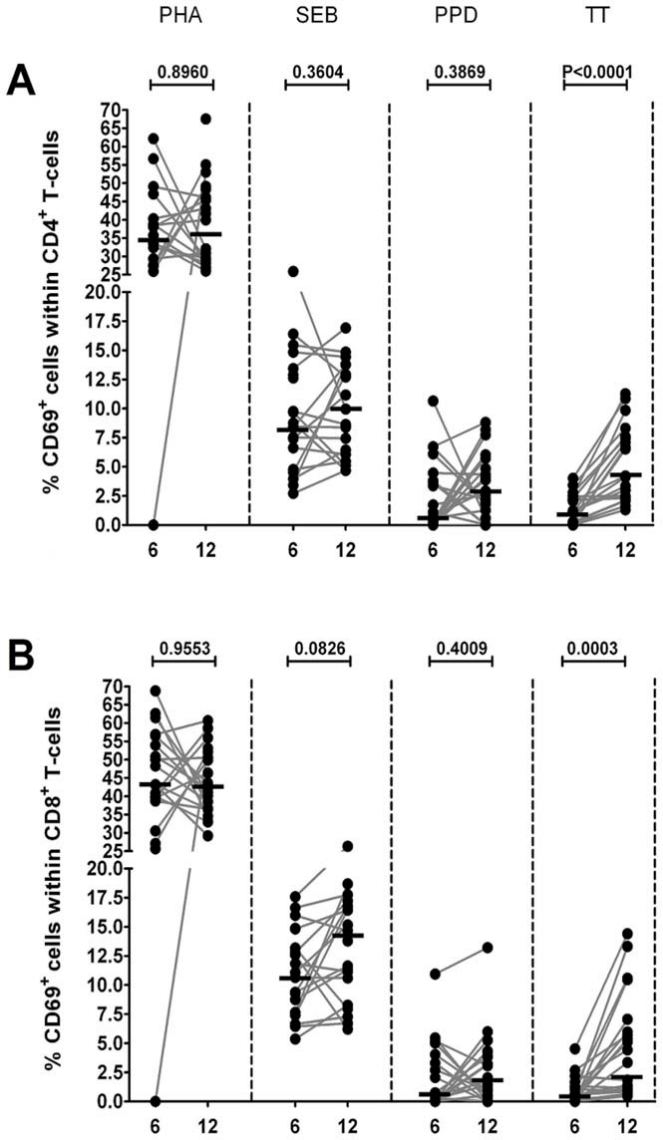
Percentages of CD69 expressing T-cells upon in vitro stimulation. Frequency of responding CD69^+^ T-cells, among CD4^+^ T-cells (**A**) and CD8^+^ T-cells (**B**), in peripheral blood samples from infants (n = 20) at 6 and 12 months of age after *in vitro* stimulation with PHA, SEB, PPD and TT. Horizontal lines represent the median percentage. The p-values were calculated using the Wilcoxon signed ranks test for paired values.

In response to PMA, all cells reached the maximum level of activation as all CD4^+^ and CD8^+^ T-cells were 100% positive for CD69^+^ (data not shown). As shown in Figure 2, the percentages of CD69^+^-expressing CD4^+^ and CD8^+^ T-cells were significantly increased after stimulation with TT in infants aged twelve months when compared to those aged six months, whereas no differences was detected in CD69^+^-expression in response to the other triggers.

### Intracellular cytokine production in CD4^+^ en CD8^+^ T-cell subsets in healthy infants: age related differences

**Figure 3:**
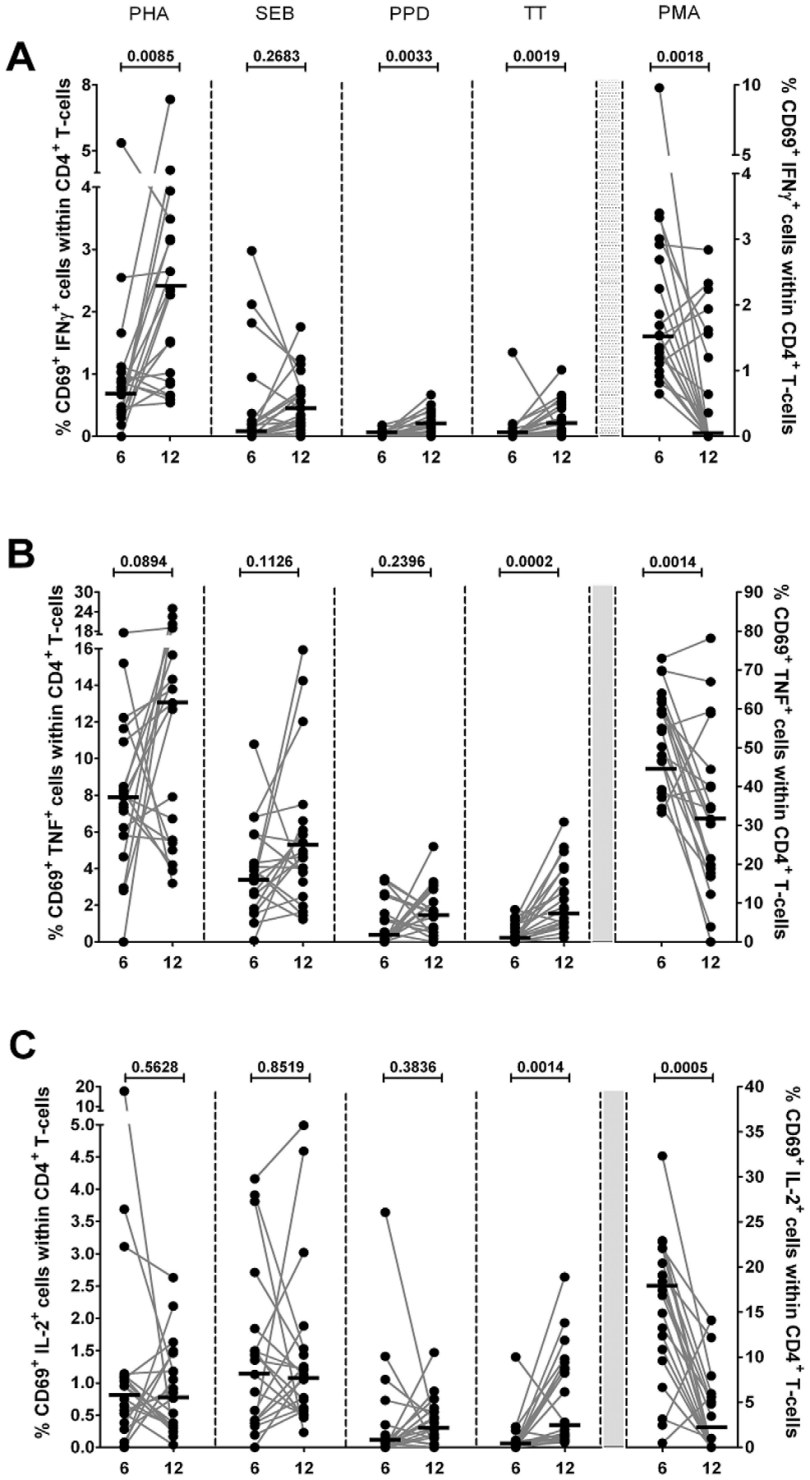
Multiparameter flow cytometric detection of cytokine expression in CD4^+^ T-cells of infants at 6 and 12 months of age. Frequencies of circulating IFN-γ (**A**), TNF-α (**B**), and IL-2 (**C**) secreting cells among CD69^+^CD4^+^ T-cells from infants (n = 20) at 6 and 12 months of age after *in vitro* stimulation with PHA, SEB, PPD, TT, and PMA. The percentages of cytokines expressing cells were measured within lymphocytes electronically gated for CD69^+^CD3^+^CD8^−^ cells. Horizontal lines represent the median percentage. The p-values were calculated using the Wilcoxon signed ranks test for paired values.

**Figure 4:**
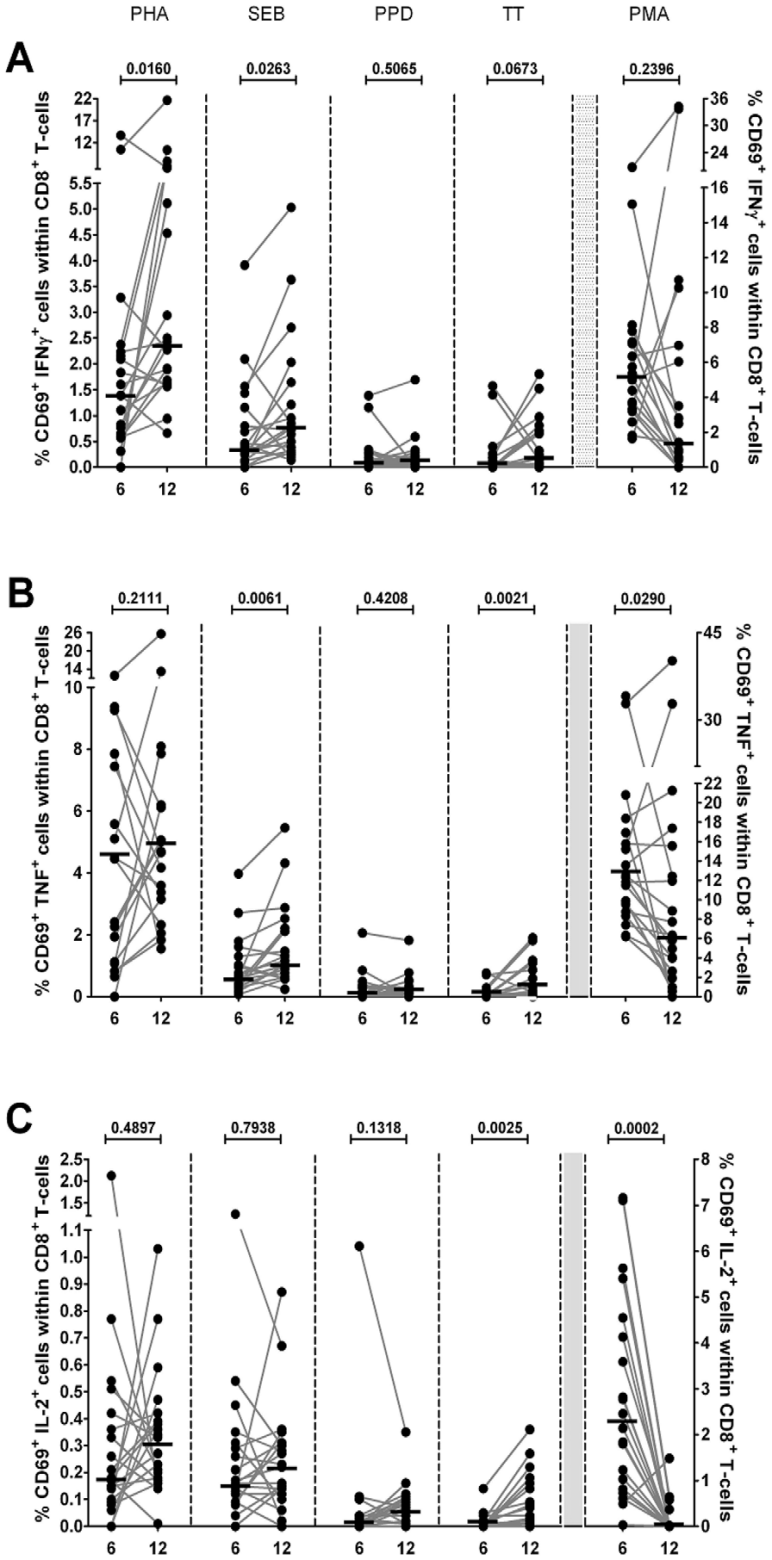
Multiparameter flow cytometric detection of cytokine expression in CD8^+^ T-cells of infants at 6 and 12 months of age. Frequencies of circulating IFN-γ (**A**), TNF-α (**B**), and IL-2 (**C**) secreting cells among CD69^+^CD8^+^ T-cells from infants (n = 20) at 6 and 12 months of age after *in vitro* stimulation with PHA, SEB, PPD, TT, and PMA. The percentages of cytokines expressing cells were measured within lymphocytes electronically gated for CD69^+^CD3^+^CD8^+^ cells. Horizontal lines represent the median percentage. The p-values were calculated using the Wilcoxon signed ranks test for paired values.

Results of the analyses are displayed in Figures 3 and 4. A significantly higher percentage of IFN-γ producing cells was observed at the age of twelve months after stimulation with PHA, PPD, and TT in CD4^+^ T-cell subsets, and after stimulation with PHA and SEB in CD8^+^ T-cell subsets. The percentage of TNF-α producing cells was higher at twelve months of age after stimulation with PHA, and TT in CD4^+^ T-cell subsets, and after stimulation with TT in CD8^+^ T-cell subsets. For both CD4^+^ and CD8^+^ T-cells, significantly higher percentages of IL-2 producing cells were found in infants aged twelve months after stimulation with TT. The percentages of IL-2, IFN-γ, and TNF-α producing cells were lower after stimulation with PMA at the age of twelve months as compared to six months for CD4^+^ T-cell subsets. In CD8^+^ T-cell subsets, significantly lower percentages of IL-2 and TNF-α producing cells were observed, whereas no significant difference was found in the percentage of IFN-γ producing cells. No significant differences were observed after stimulation with PPD for CD8^+^ T-cell subsets.

## Discussion

In this report we successfully applied the FCB method to assess Ag-specific T-cell cytokine patterns in limited blood samples in infants from whom small amounts of blood are available. Using the modified FCB method we demonstrate an increase in the intracellular production of IL-2, IFN-γ, and TNF-α cytokines in infants aged twelve compared to infants aged six months when stimulated with PHA, SEB, PPD, and TT. Only stimulation with PMA resulted in an overall lower intracellular cytokine production. The results were reproducible in CD4^+^ and CD8^+^ T-cell subsets. Based on the consistency of our results we are convinced that the FCB method is a promising tool to measure intracellular cytokine production when limited amounts of blood are available, and may prove to be a valuable aid in studying immune regulation in infants.

The absolute number of the T-cell subpopulations at the age of six and twelve months of the infants in our study corresponds well with previous literature [14]. The CD69^+^ antigen expression level was assessed, because this activation antigen is expressed early by T-cells following exposure to stimuli [15]. Based on the expression frequency of CD69^+^, we initially assessed the percentage of mitogen- (PMA, PHA) and antigen- (SEB, PPD, TT) responding CD4^+^ and CD8^+^ T-cells in peripheral blood from infants at the age of six and twelve months. CD69^+^ expression was shown in both CD4^+^ and CD8^+^ T-cells after stimulation with the different mitogens and antigens. The expression level of CD69^+^ in response to PMA in CD4^+^ T-cells as well as CD8^+^ T-cells was 100% for this activation marker. In our opinion, the CD69^+^ antigen expression level is a good indicator of overall response to stimulation and further substantiates our conviction that we have successfully applied the FCB-method for measuring multicytokine responses.

Krutzik and Nolan [8] described several technical considerations that have to be kept in mind while applying the FCB method. One of these considerations is the potential impact of cell numbers on robustness of the staining. Another consideration is the importance of optimizing the dye for each cell type as high concentrations of dye may lead to unstained samples showing significant labelling. In our study, in order to reduce the risk of these potential problems, we washed the samples thoroughly and used smaller quantity of dye. Also, we used different dyes than the ones used by Krutzik and Nolan. Our procedure was optimized by using small amounts of the Pacific Blue and Pacific Orange dye. The novelty of this FCB method is that the different barcoded samples are separated into different gaps, which allows the different labels to gate separately during the FACS analysis. In this study, it was clearly shown that unstained samples are gated separately.

Some studies have observed age-related differences in intracellular cytokine production in healthy children. Most of these studies used PMA as a stimulant and suggest that the Th1-type T-cell cytokine production in young children or neonates is lower than in adults [9]–[12]. These data suggest that the Th1-response is impaired in infants compared to older children and adults, and that progressive maturation occurs with age [10]. Other studies used PHA and/or SEB to stimulate either PBMCs or T-cells *in vitro* and found similar results [16]–[23]. Our study shows a significantly higher Th1-type cytokine production in CD4^+^ T-cell subsets after stimulation with PHA, PPD, and TT at the age of twelve months compared to six months, and significantly higher intracellular cytokine production in CD8^+^ T-cell subsets after stimulation with PHA and SEB. Our findings do not support an impaired intracellular cytokine production in infants aged six months compared to twelve months when stimulated with PMA, but because the other more specific stimuli led to more intracellular Th1-type cytokine production, we overall support the view that Th1-responses are impaired in young infants and that they mature with age.

To our knowledge, this is the first study to measure intracellular cytokine responses in healthy infants in response to PPD. PPD, or tuberculin, is a large protein with many antigenic properties used as a diagnostic agent for infection with Mycobacterium tuberculosis. In tuberculosis, primarily CD4^+^ T-cell subsets are involved in the amplification of an immune response by secreting several cytokines [24]. We conducted this study among 20 healthy Dutch infants, who are from a non-endemic country for tuberculosis and are not immunized with the Bacillus Calmette-Guérin (BCG) vaccine. Our study showed detectable levels of PPD-induced intracellular IL-2, IFN-γ, and TNF-α production for both CD4^+^ and CD8^+^ T-cell subsets. A significantly higher IFN-γ-production in CD4^+^ T-cell subsets was observed at the age of twelve months compared to six months. It has been shown that individuals with tuberculosis have a strong Th1-response, and IFN-γ is essential for protection against tuberculosis [25], [26]. The best explanation for the intracellular cytokine response in non-infected infants not vaccinated with the BCG in our view is molecular mimicry.

Information on tetanus-specific T-cell responses is mostly limited to studies in mice and adults [27], [28]. In these studies, both Th1- and Th2-response have been implicated in vaccine induced protection. A study by Rowe et al. [29] assessed the time-dependent changes of multiple cytokine responses to TT antigen in infants in the first year of life. They measured antigen specific cytokine production (IL-4, IL-5, IL-6, IL9, IL-10, IL-13 and IFN-γ) in the supernatants of PBMCs at the age of two, four, six, and twelve months. Statistically significant age-related differences were found for IL-4, IL-9, IL-13, and IFN-γ production. The Th2-responses were relatively stable between six and twelve months; however, the IFN-γ production was significantly lower during this period. This indicates an early and persistent Th2-response, in contrast to a more delayed maturation of the Th1-component of the vaccine response during infancy. In contrast to the delayed maturation of the Th1-component of the vaccination response in the previous mentioned study by Rowe, we found an overall significantly higher cytokine production in infants aged twelve months compared to infants six months of age. An explanation for our findings might be that in the Netherlands we give a tetanus booster at the age of eleven months, while no booster was given until eighteen months in the study by Rowe.

In summary, based on our results we believe that the FCB method can be a valuable aid in studying immune responses in infants and because only limited amounts of blood are needed, it will allow for extended studies in larger populations during early life. We also show that the Th1-responses are overall impaired at the age of six months when compared with twelve months.
